# Source identification and risk assessment of potentially toxic elements in surface soil of townships in the northwest Hebei region

**DOI:** 10.1371/journal.pone.0353293

**Published:** 2026-08-28

**Authors:** Zhanbin Wang, Daokun Chen, Xinbin Li, Ke Yang, Dongxiang Jiang, Donglin Wang, Liannan Shang, Yuekun Wang

**Affiliations:** 1 Xi’an Mineral Resources Survey, China Geological Survey, Xi’an, China; 2 State Key Laboratory of Deep Earth Exploration and Imaging, School of Geophysics and Information Technology, China University of Geosciences, Beijing, China; 3 Qinling--Loess Plateau Transition Zone Observation and Research Station for Coupling of Soil and Water Elements and Conservation of Biological Resources, Tongguan, China; University of Westminster - Regent Street Campus: University of Westminster, UNITED KINGDOM OF GREAT BRITAIN AND NORTHERN IRELAND

## Abstract

Soil environmental quality in rural areas is not only crucial to the implementation of the rural revitalisation strategy, but also directly impacts the smooth progress of integrated urban-rural development. However, it faces an increasing risk of contamination from PTEs. Consequently, systematically identifying sources of contamination and assessing their ecological and health risks is essential to ensuring soil safety and sustainable development in rural areas. The results showed that the mean content of eight PTEs in the study area was lower than the background value of the soil environment in Hebei Province only for the elements As, Pb, Zn and Hg, and the exceeding rate of Cr, Cu, Cd and Ni was higher than 55%. APCS-MLR model identified five categories of pollution sources with the following sources and contributions: industrial pollution sources (47.1%), transportation sources (24.3%), natural sources (13.8%), agricultural sources (3.8%), and atmospheric deposition sources (11.0%). Ecological risks are mainly attributed to Cd and Hg, with high-risk areas concentrated in the central part of the study area. Probabilistic health risks indicate that adult and pediatric THI values are within the safe range at the 95% confidence interval and that 16.98% children have some significant carcinogenic risk. Source contribution to health risk indicates that industrial and transportation sources are important sources of control for cancer risk, yet elemental As are less enriched, so there is no need to pay much attention to the impact of natural sources on health risk. In conclusion, this study provides a reference for risk prevention and potential source identification of PTEs in the top soil of the township.

## 1. Introduction

Soil, as a fundamental component of terrestrial ecosystems, underpins nearly all human socioeconomic activities [1]. However, rapid industrialization and urbanization have led to the substantial accumulation of PTEs in soils, exceeding their natural self-purification capacity and resulting in widespread environmental contamination [2,3]. According to the 2023 China Ecological and Environmental Status Bulletin [4], although the escalating trend of soil pollution has been partially mitigated—with national arable land averaging a quality grade of 4.76 and high-quality soils accounting for 31.24%—PTEs contamination remains a critical environmental challenge requiring immediate remediation. PTEs exhibit high environmental persistence, impairing crop productivity and posing severe health risks through bioaccumulation in the food chain [5–8]. Nevertheless, such macroscopic assessments may fail to resolve localized contamination hotspots at the township scale, where intensive industrial and agricultural activities often converge. This constitutes a critical knowledge gap, as no existing study has systematically assessed PTEs contamination risks specifically at the township level using high-density sampling.

While existing research has extensively investigated PTE risks at large spatial scales (e.g., county-level and above) and localized environments (e.g., watersheds, mining areas), township-level assessments remain notably underrepresented [9–11]. Zhangjiakou is an important vegetable production base for Beijing, with abundant agricultural production. Previous research has primarily focused on soil pollution in the Beijing-Tianjin-Hebei region [12] and counties within Hebei Province [13,14]. However, large-scale studies have often prioritized macro-level control, resulting in insufficient sampling density and precision, which limits the practical applicability of the findings. Given the increasing role of townships in industrial relocation amid rapid socioeconomic transitions, these areas face heightened exposure to PTEs from both industrial emissions and conventional agricultural practices, leading to progressive toxicant accumulation in arable soils. However, to date, no research has conducted high-density, township-scale sampling in this region, leaving a clear knowledge gap regarding localized contamination hotspots. Thus, a systematic and quantitative assessment of soil environmental quality at the township scale can provide local authorities with precise insights into contamination risks, enabling targeted control and isolation measures. Conducting high-density sampling at the township level is a prerequisite for precise research. Additionally, it offers critical guidance for ensuring regional crop production safety.

Currently, many scholars have concluded that the contamination of soil potentially toxic elements is affected by both natural and anthropogenic factors [15], and therefore the multi-perspective characterization of risk is the main trend in assessing the hazard of soil potentially toxic elements in the region [16]. Using geostatistical analysis to characterize differences in the spatial distribution of elements and combining Pearson correlation, cluster analysis and principal component analysis to qualitatively assess the sources of pollutants. While this characterization method is limited to qualitative source analysis, receptor modeling offers a quantitative approach to source apportionment by leveraging the physicochemical properties of pollutants in both emission sources and environmental receptors. This enables not only source identification but also precise quantification of contribution rates, thereby facilitating evidence-based prioritization of pollution control measures [17,18]. The APCS-MLR receptor model can be used to quantitatively resolve the possible sources of contaminants and address the limitations of qualitative analysis [19]. Hence, this study combines APCS-MLR modeling and Pearson correlation to systematically assess the sources of pollutants. Potential ecological risk method is used to quantify the degree of contamination of soil with potentially toxic elements, which is characterized by the visual characterization of the level of regional ecological risk status [20].

Traditional deterministic health risk assessment is a quantitative approach; however, exposure risk varies due to the variability in individual physiological conditions. The application of Monte Carlo simulation for probabilistic health risk assessment can reduce this uncertainty and thereby improve the accuracy of the risk assessment results.[21,22]. By establishing a source-oriented health relationship between potential toxic elements and pollution sources in soil and health risks, its based on probability health risk combined with the APCS-MLR model to quantify the contribution of potential toxic elements from different sources in soil [23–25]. Effective information is provided for regional health risk control and prevention, thereby determining the priority of health hazards. In summary, while previous studies have either relied on qualitative source analysis or employed deterministic health risk assessment in isolation, none has integrated quantitative source apportionment (APCS-MLR) with probabilistic health risk assessment (Monte Carlo) at the township scale. This study explicitly addresses these methodological gaps by establishing a source-oriented health risk assessment framework that links specific pollution sources to their probabilistic health impacts.

The main objectives of this study are as follows: (1) Pearson correlation and APCS-MLR model were used to qualitatively and quantitatively identify pollution sources and their contribution. (2) Potential ecological risk index (RI) to assess soil contamination characteristics and risks. (3) Probabilistic health risk assessment using Monte Carlo simulation and prioritization of control factors by quantifying health risks in terms of source contributions.

## 2. Materials and methods

### 2.1. Study area and sample collection

Zhangjiakou is situated in the north-west of Hebei Province, at the junction of two major tectonic units: the Inner Mongolia–Greater Khingan Fold System and the Sino-Korean Craton. Strata are widely exposed, ranging from the Archaean to the Cenozoic. The terrain slopes from west to east, with the Yin Mountains running through the centre of the city, dividing it into two parts: the area above the dam (at an altitude of approximately 1,400 metres) and the area below the dam (at an altitude of 1,000–2,000 metres). The Yanghe and Sanggan Rivers flow from east to west through the urban area, emptying into the Guanting Reservoir. The soil on the upper dam is predominantly chernozem, with relatively poor fertility; in the intermontane basins of the lower dam, the soil is mainly brown soil, which is more fertile and suitable for cultivation. The region has a temperate continental monsoon climate. The climate in the upper dam area is cold, with a frost-free period of only 90–110 days; the lower dam area enjoys better thermal conditions, with significant diurnal temperature variations and a longer frost-free period. Precipitation within the region is low and extremely unevenly distributed; annual precipitation in the upper dam area is only 330–400 millimetres, whilst in the lower dam area it can reach 400–500 millimetres.

**Fig 1:**
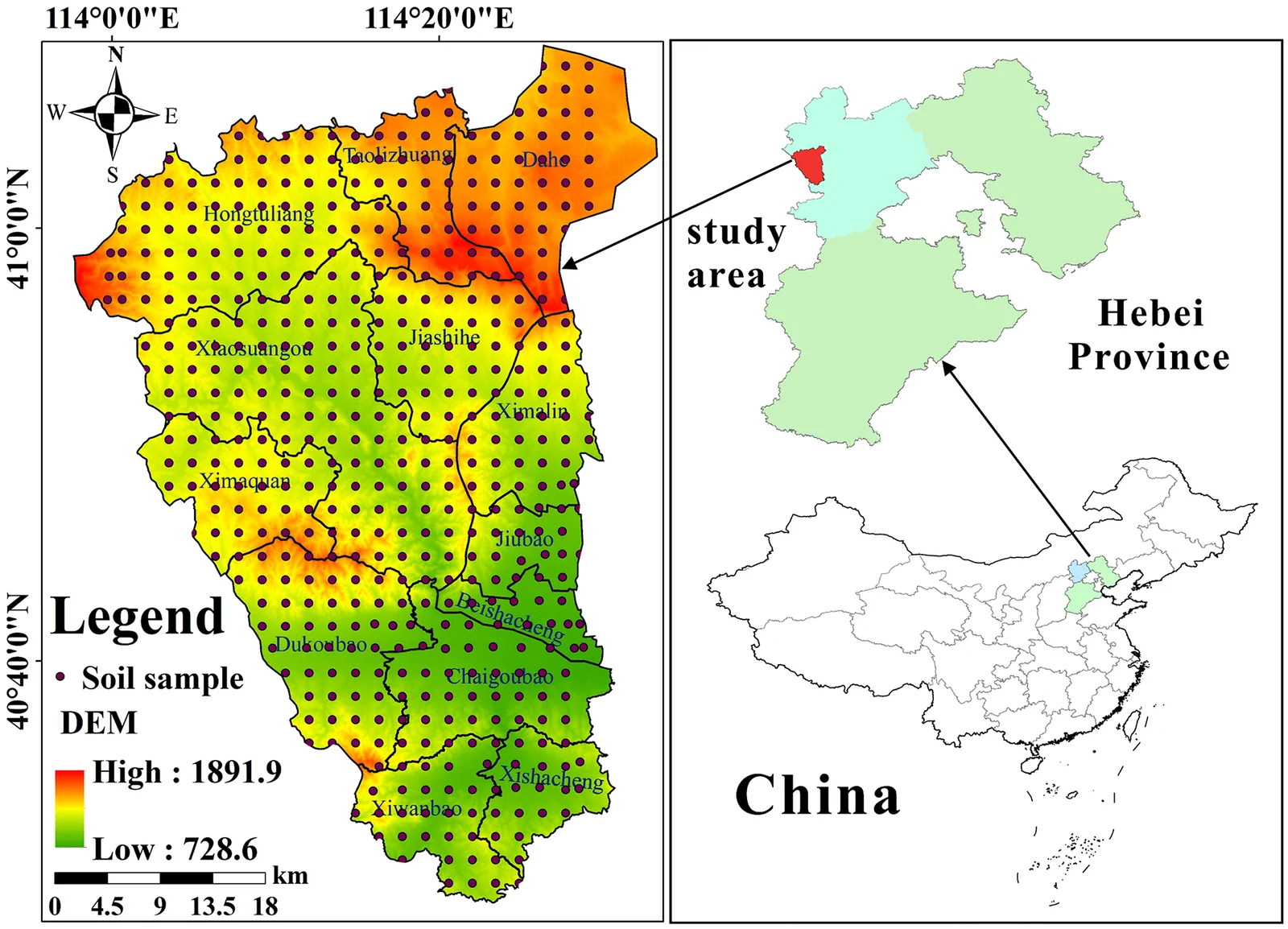
Location and sampling map of the study area. The base map was sourced from Tianditu (https://www.tianditu.gov.cn**).**

The study area is located in the northwestern part of Zhangjiakou City and involves a total of 13 townships (including Hongtuliang Township, Taolizhuang Township, Jiashihe Township, Xiaosuangou Township, Xiamaquan Township, Dahe Township, Ximalin Township, Jiubao Township, Beishacheng Township, Chaigoubao Township, Xishacheng Township, Xiwanbao Township, and Dukoubu Township).Sample sites in the study area were laid out in accordance with the “Specifications of Multi-Purpose Regional Geochemical Surveys” (DZ/T 0258–2014) [26]. As shown in Fig 1, 520 surface soil samples (depth 0–20 cm) were collected at fixed points using GPS, and four sub-sampling points were collected within a 20-meter radius of each fixed point. Equal amounts were collected uniformly and combined into one sample to ensure that the samples were representative. The sample volume of each sample was at least 1 kg, and the sampling point number, coordinates and other basic information were recorded. After removing grass, leaves, roots and other impurities through a 2 mm nylon sieve, the samples were stored in kraft paper and sent to the laboratory for analysis.

### 2.2. Chemical analysis

Samples are dried at a temperature of less than 60°C before being sent to the laboratory for assay. Samples were processed in a non-polluting ball mill. The samples were processed to a particle size of 0.074 mm. The loss rate of sample preparation was controlled at 0 ~ 4.25%, and the shrinkage error was less than 2.96%, which was in accordance with the requirements of technical specifications. As and Hg were determined by digestion with aqua regia before the determination, and other elements were determined by digestion with HNO3-HCl-HF-HClO4 before the determination. As was determined by hydride generation-atomic fluorescence spectrometry (AFS); Hg was determined by cold vapor-atomic fluorescence spectrometry (CV-AFS); Cr and Mn were determined by inductively coupled plasma-emission spectrometry (ICP-OES); and the content of Ni, Cu, Cd, Pb, and Zn were measured by inductively coupled plasma-mass spectrometry (ICP-MS). The quality control of the determination of each element was controlled by the method of national level standard substance, and the results of each element were within the tolerance range, and the results were in accordance with the quality control requirements for sample analysis. The recovery rates for all elements were greater than 90%, with an overall recovery rate of 97.88%. Analyses were conducted using GBW series national reference standards (high, medium and low concentrations) to calculate and evaluate the accuracy and precision of the analyses, for the purpose of quality control in regulatory testing. The accuracy of the analytical methods for each element was within 0.11, and the relative standard deviation (RSD) for precision was within the 10% limit required for quality control.

### 2.3. APCS-MLR source identification model

The Absolute Principal Component Score-Multiple Linear Regression (APCS-MLR) receptor model combines Absolute Principal Component Scores (APCS) derived from Principal Component Analysis (PCA) with Multiple Linear Regression (MLR) to quantitatively apportion pollution sources [27]. This model assumes that the total pollutant concentration at a receptor site represents a linear superposition of contributions from all potential sources, thereby enabling quantitative source attribution [28]. In the APCS-MLR framework, the elemental concentration dataset is first standardized and transformed into APCS, which serve as predictor variables in MLR, while measured concentrations of PTEs are treated as the response variable. By resolving the MLR equation, the model quantifies the fractional contribution of each pollution source, addressing a critical limitation of conventional PCA—its inability to quantify source-specific contributions beyond qualitative identification [29]. The computational procedure involves the following steps:


$$\begin{array}{c} C_{i}=b_{i}+\sum_{k=1}^{p}b_{ki}\times {\text{APCS}}_{k} \end{array}$$ (1)

$$\begin{array}{c} Pc_{i}=\frac{|b_{ki}\times \text{AP}{\overset{―}{\text{CS}}}_{k}|}{\sum_{k=1}^{p}\left|b_{ki}\times \text{AP}{\overset{―}{\text{CS}}}_{k}\right|} \end{array}$$ (2)

where C_i_ is the measured concentration of soil potentially toxic elements element i, b_i_ is the constant term of the regression equation, b_ki_ is the regression coefficient of pollution source k to potentially toxic elements element i, APCS_k_ is the absolute principal factor score of factor k. b_ki_ × APCS_k_ denotes the amount of source k contribution to C_i_; The average of all samples b_ki_ × APCS_k_ represents the average absolute contribution of source k to C_i_; Pc_i_ is the contribution of element i to source k.

### 2.4. Ecological and health risk assessment of PTEs in soil

#### 2.4.1. Potential ecological risk index.

The potential ecological risk index (RI) method assesses the negative impact of pollutants on the ecosystem from a toxicological perspective [30]. Potential ecological risk levels in the environment were determined by statistically evaluating RI for various components and potential ecological risk coefficients (E^i^_r_) for each element [30]. Calculation formulae are as follows:


$$\begin{array}{c} RI=\sum_{i=1}^{n}E_{i}=\sum_{i=1}^{n}T_{i}\times \frac{C_{i}}{B_{i}} \end{array}$$ (3)

Where RI is the potential ecological risk index of various heavy metals at the sampling site; E_i_ is the potential ecological risk coefficient of element i; T_i_ is the toxicity coefficient of element i (40, 30, 10, 5, 5, 5, 5, 2, and 1 for Hg, Cd, As, Ni, Pb, Cu, Cr, and Zn elements, respectively) [31]; C_i_ is the measured content of element n in the soil; B_i_ is the soil geochemical background value of the element in Hebei Province [32]. Its potential ecological risk coefficient (E_i_) and potential ecological risk index (RI) grading criteria are shown in supporting data file S1 Table in S1 File.

#### 2.4.2. Probabilistic health risk assessment.

The human health risk assessment model, proposed by the U.S. Environmental Protection Agency (EPA), is designed to quantify and evaluate the carcinogenic and non-carcinogenic risks posed by potentially toxic elements to human populations [33]. By examining three exposure pathways—ingestion, inhalation, and dermal contact—for both adults and children, and integrating Monte Carlo simulation, this model probabilistically assesses the carcinogenic and non-carcinogenic risks associated with potentially toxic elements [34]. The calculation formulas for this assessment model are as follows:


$$\begin{array}{c} ADD_{ing}=C_{s}\times \frac{IngR\times EF\times ED}{BW\times AT}\times 10^{-6} \end{array}$$ (4)

$$\begin{array}{c} ADD_{inh}=C_{s}\times \frac{InhR\times EF\times ED}{PEF\times BW\times AT} \end{array}$$ (5)

$$\begin{array}{c} ADD_{der}=C_{s}\times \frac{AF\times SA\times ABS\times EF\times ED}{BW\times AT}\times 10^{-6} \end{array}$$ (6)

In the above formula, ADD_ing_、ADD_inh_ and ADD_der_ are the average intake by oral、respiratory and dermal routes, respectively. C_s_ is the actual measured concentration of potentially toxic elements. Details of the remaining parameters (IngR, InhR, EF, ED, BW, AT, PEF, SA, AF, and ABS) are given in the supporting data file S2 Table in S1 File.


$$\begin{array}{c} HI=\sum HQ_{i}=\sum \frac{ADD_{ing}+ADD_{inh}+ADD_{der}}{RfD_{i}} \end{array}$$ (7)

$$\begin{array}{c} CR=\sum CR_{i}=\sum (ADD_{ing}+ADD_{inh}+ADD_{der})\times SF_{i} \end{array}$$ (8)

Hazard Index (HI) represents the cumulative non-carcinogenic risk of all PTEs in soil. An HI value <1 indicates negligible non-carcinogenic health risks from soil PTEs [35]. Carcinogenic risk (CR) denotes the total carcinogenic risk index of soil PTEs. When CR < 10^−6^, the cancer risk for local residents is considered minimal; whereas CR > 10^−4^ suggests a significantly increased probability of developing cancer [36]. The slope factor (SF) refers to the cancer risk reference dose, while the Reference Dose (RfD) for ingestion (RfD_i_) represents the element-specific reference dose for a particular exposure pathway. Detailed information on SF and RfD values for different PTEs exposure pathways in soil is provided in S3 Table in S1 File.

Monte Carlo simulation is a computational algorithm that employs probabilistic random sampling based on mathematical formulations to derive approximate solutions to complex problems [37]. This method has been widely adopted by researchers for uncertainty analysis in risk assessment studies [38]. By integrating Monte Carlo simulation with human health risk assessment models, we can effectively characterize the probabilistic health risks posed by PTEs in soil while minimizing potential overestimation or underestimation biases arising from individual variability [39].In this study, we performed 10,000 iterations of Monte Carlo simulations using Crystal Ball software with a 95% confidence interval [40]. Detailed information on other parameters used in the simulation is provided in S4 Table in S1 File.

### 2.5. Statistical analyses

In this study, comprehensive data analysis was performed using multiple statistical platforms: (1) Descriptive statistics and Pearson correlation analysis of surface soil PTEs were conducted using Origin Pro 2021 with subsequent generation of relevant graphical representations; (2) Ecological risk assessment of soil PTEs was calculated using Microsoft Excel 2021; (3) Normality distribution testing and source apportionment calculations via the APCS-MLR receptor model were implemented in IBM SPSS Statistics 26; (4) Probabilistic health risk assessment was performed through Monte Carlo simulation using Crystal Ball software.

## 3. Results and discussion

### 3.1. Soil PTEs content characteristics and spatial distribution

Table 1 displays the descriptive statistical findings of PTEs in the research area’s soils. As, Cd, Cr, Cu, Ni, Pb, Zn, and Hg had mean values of 4.99, 0.11, 93.81, 31.1, 43.7, 18.3, 72.1, and 0.014 mg/kg, respectively. The only elements with mean values below the Hebei Province soil environmental background values were As, Pb, Zn, and Hg. All eight PTEs had exceedance rates higher than 0, suggesting that each element was somewhat enriched. Notably, the rates of Cr, Cu, Cd, and Ni exceedance surpassed 50%, indicating a substantial impact from human activity. The variability and dispersion of PTEs in soils were evaluated using the coefficient of variation (CV). Cu, Ni, and Hg showed substantial variability in the CV results, suggesting that external variables had a significant impact on these components.

**Table 1:** Descriptive statistics of PTEs in soils of the study area (mg/kg).

Elements	As	Cd	Cr	Cu	Ni	Pb	Zn	Hg
Min	0.63	0.032	22.0	6.0	10.0	6.31	27.0	0.004
Max	17.0	0.500	312.0	85.0	193.0	32.0	305.0	0.180
Mean	4.99	0.110	93.81	31.1	43.7	18.3	72.1	0.014
SD	1.98	0.044	44.9	16.0	25.6	3.98	27.7	0.014
CV(%)	39.81	39.79	47.91	51.41	58.55	21.77	37.05	98.17
Skewness	1.14	3.31	1.23	0.83	1.48	−0.09	1.85	6.99
Kurtosis	4.1	22.52	1.71	−0.06	3.55	0.33	10.77	66.48
Exceedance rate (%)	0.57	59.92	66.6	64.62	59.54	18.32	36.07	3.05
BV	13.6	0.094	68.3	21.8	30.8	21.5	78.4	0.036

Note: BV: Background value of Hebei Province.

**Fig 2:**
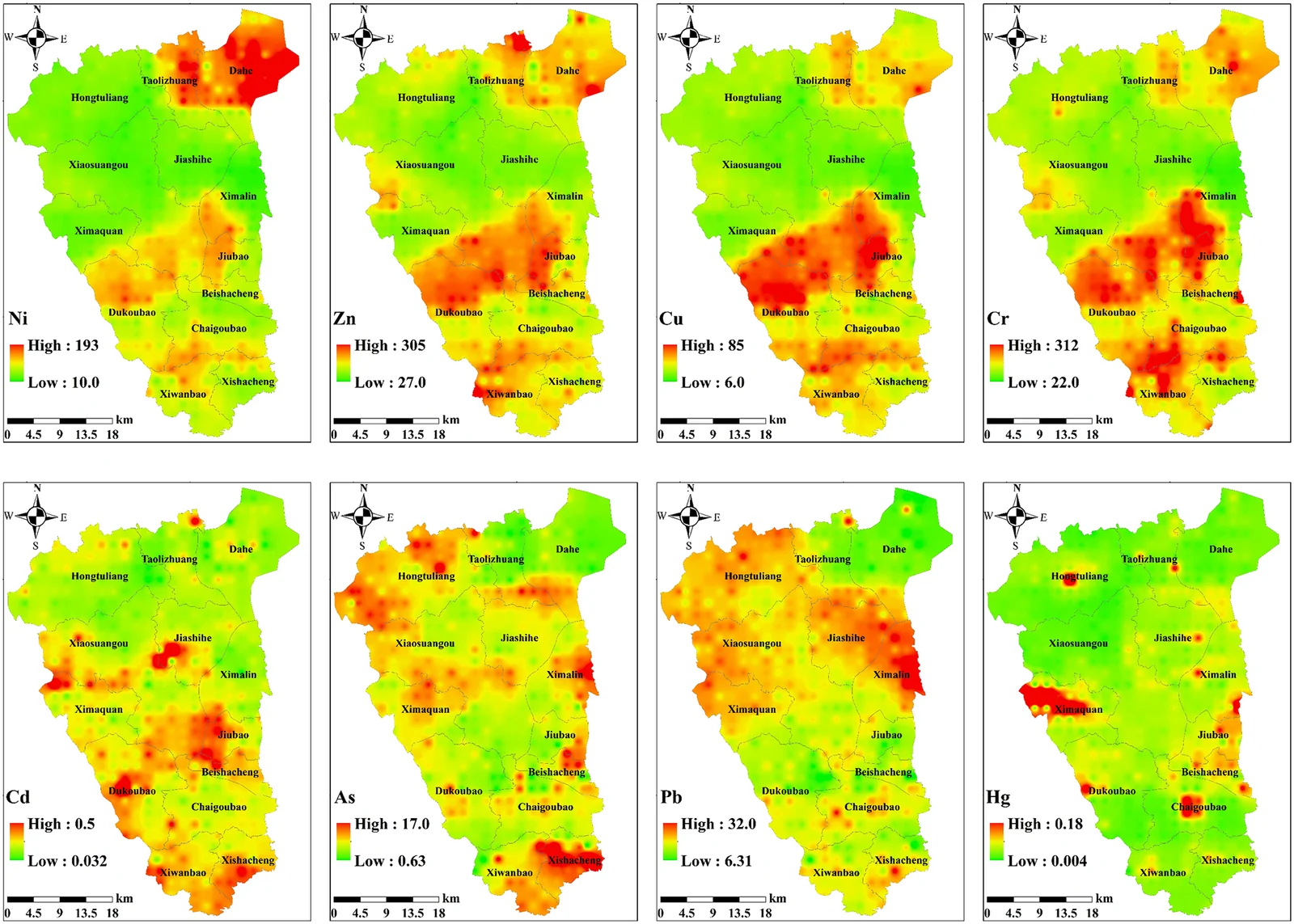
Spatial distribution characteristics of eight PTEs. The base map was sourced from Tianditu (https://www.tianditu.gov.cn).

The spatial distribution characteristics of PTEs in the study area are illustrated in Fig 2. Element As exhibits a relatively dispersed spatial pattern, generally forming patchy clusters. Higher anomaly points are observed in the Xishacheng Township, with varying degrees of accumulation detected across all townships. Anomalies of Cd are predominantly concentrated in the southwestern region, forming localized point clusters that coalesce into strip-like distributions. All townships show certain levels of Cd accumulation, albeit with considerable spatial variability. Elements Cr, Cu, Ni, and Zn demonstrate broadly similar spatial distribution patterns, suggesting potential common sources. Hg anomalies are mainly concentrated in the central part of the study area, where elevated concentrations affect multiple townships, indicating a regionally enriched pattern. In contrast, significant Pb accumulation is absent only in Dahe Township and Taolizhuang Township, while other regions exhibit varying degrees of enrichment, displaying distribution characteristics distinct from those of the other elements. Overall, the spatial patterns of high anomalies for these eight heavy metals suggest potential pollution risks in the region, likely attributable to external inputs. These findings provide a basis for further source identification research.

### 3.2. APCS-MLR source identification results

**Fig 3:**
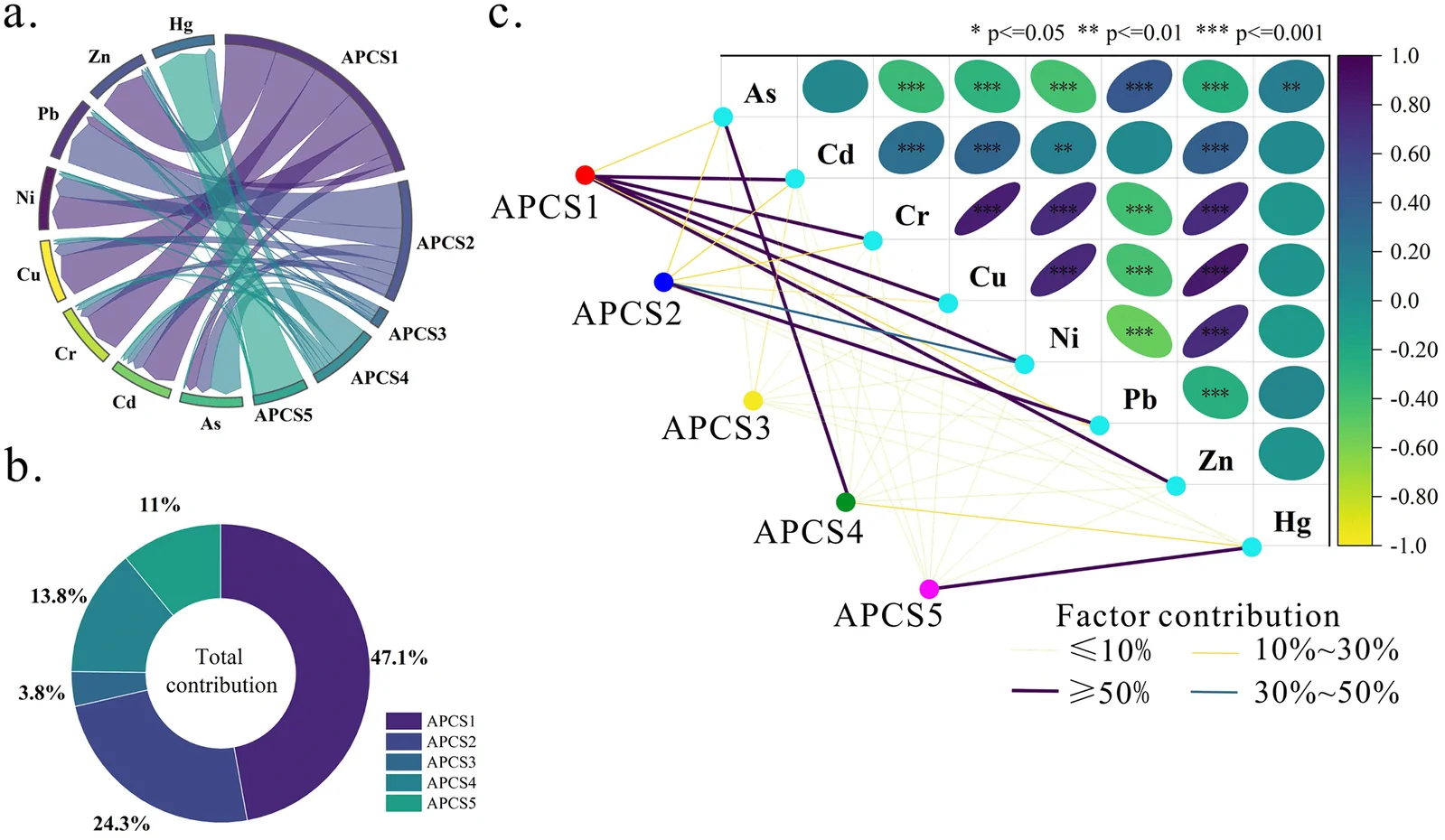
Map of correlation and source contribution of PTEs in the study area; a – contribution of each source of PTEs; b – percentage of each source factor; c – correlation between the content of PTEs and source factors.

This study employed the APCS-MLR model to quantitatively assess sources and contribution rates of PTEs in soils. Initial data normalization using the KMO-Bartlett sphericity test (KMO = 0.811, p < 0.001) confirmed dataset suitability for factor analysis with statistically significant inter-element correlations [41]. PCA of eight PTEs identified five significant components (eigenvalues >1) explaining 92.95% of total variance after Kaiser normalization and varimax rotation. The PCA results are shown in S5 Table and S6 Fig in S1 File. Subsequent MLR analysis incorporating PTEs concentrations and absolute principal component scores (APCS) demonstrated model reliability, with all fitted coefficients exceeding 0.8. The contribution rates of potential sources are presented in Fig 3b: APCS1 (47.1%), APCS2 (24.3%), APCS3 (3.8%), APCS4 (13.8%), and APCS5 (11.0%). The significant differences in contribution rates among sources indicate that the spatial heterogeneity of PTEs may be influenced by variations in input quantities from different pollution sources. Key findings are summarized as follows:

Fig 3a reveals APCS1 as the predominant source for Zn (84.9%), Cr (72.0%), Cu (70.6%), Cd (59.8%), and Ni (57.5%). The marked variability of these elements indicates significant anthropogenic enrichment. Correlation analysis demonstrated partial elemental homology, with Cd showing weaker associations with Zn, Cr, Cu, and Ni (Fig 3c), this result is consistent with the spatial distribution shown in Fig 2, suggesting distinct Cd sources. Previous studies confirm Cr and Ni as reliable industrial tracers [42,43], while Zn, Cu, and Cd concentrations are characteristically elevated in industrial/construction soils compared to agricultural soils [44]. These findings collectively identify APCS1 as an industrial pollution source.

APCS2 primarily contributes to Pb (76.7%) and secondarily to Ni (33.3%) (Fig 3a). The low variability and relatively uniform spatial distribution of Pb suggest limited external influences, likely attributable to the historical legacy from leaded gasoline. Both Pb and Ni elements are recognized transportation-derived contaminants, originating predominantly from vehicular brake wear and related mechanical abrasion processes [45,46]. Therefore, APCS2 is a source of transportation pollution.

APCS3 exhibited significant contribution solely to Cd (10.4%) (Fig 3a). As shown in Fig 2, Cd is primarily concentrated in the southern region. This finding may be attributed to the low-lying terrain in the southern area, which facilitates frequent agricultural activities and consequently leads to accumulation. Cd as a well-documented agricultural tracer element, Cd predominantly originates from pesticide applications. Intensive agricultural practices in the study area, particularly increased fertilizer and pesticide use, have been shown to promote Cd accumulation in soils [47,48]. Previous studies have concluded that Cd in the Hebei Plain is affected by agricultural pollution [49], agricultural land in the Zhangjiakou region has experienced significant contamination due to the application of chemical fertilizers and pesticides [50]. Therefore, APCS3 is an agricultural source.

Fig 3a shows that the main characteristic of APCS4 is As (53.43%), a lithogenic tracer element. In this study, the average concentration of arsenic remained below background levels, with only 0.57% of sampling points exceeding the threshold. Extensive research has concluded that soil arsenic primarily originates from crustal weathering and parent material deposition [51,52]. These findings collectively confirm that APCS4 is of natural origin.

APCS5 mainly contributes 83.03% to the element Hg, and contributes a lower percentage to all other elements (Fig 3a). Hg is low in the natural background, and Hg is mainly affected by anthropogenic industrial and mining production emissions, fuel combustion, and waste incineration [53]. Based on previous research findings in a vegetable cultivation area in Wanquan District, Zhangjiakou, Hg levels are influenced by atmospheric deposition [54].Therefore, APCS5 can be recognized as a source of atmospheric deposition.

Based on the above analysis, the source identification model has identified five potential pollution sources: industrial pollution source (APCS1:47.1%), transportation pollution (APCS2:24.3%), agricultural practices (APCS3:3.8%), natural origin (APCS4:13.8%), and atmospheric deposition (APCS5:11%). These findings highlight industrial pollution as the predominant contributor to PTEs contamination, warranting prioritized control measures to mitigate ongoing environmental inputs.

### 3.3. Ecological and health risk assessment of PTEs

#### 3.3.1. Ecological risk assessment of PTEs.

**Fig 4:**
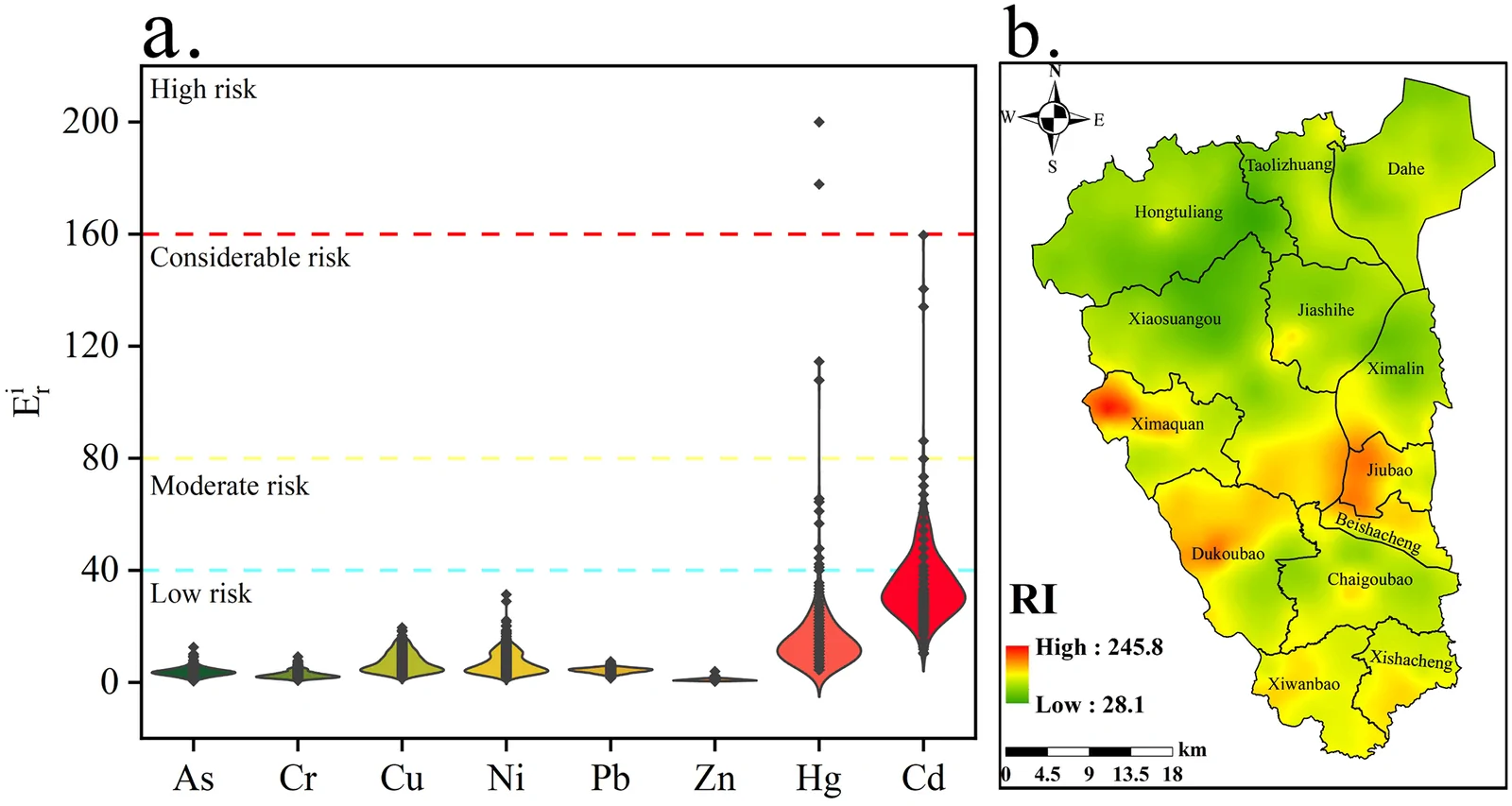
Evaluation of the potential ecological risk of PTEs in the study area; a- Violin plot of potential ecological risk coefficients for PTEs; b- Characteristics of the spatial distribution of potential ecological risk coefficients. The base map was sourced from Tianditu (https://www.tianditu.gov.cn).

Fig 4 presents the results of the ecological risk assessment for PTEs within the study area. Fig 4a illustrates the potential ecological risk coefficients of the heavy metals. The coefficients for As, Cr, Cu, Ni, Pb, and Zn fall within the slight ecological risk range, indicating minimal harm to the ecological environment. In contrast, the coefficients for Cd and Hg range from slight to very strong ecological risk. Therefore, among the eight soil heavy metals in the study area, Cd and Hg warrant particular attention regarding their ecological risk. The mean RI value in the study area is 76.8, which is substantially below 150 and falls within the slight ecological risk category, suggesting that the study area is at low risk of ecological harm. Overall, the RI values range from 28.1 to 245.8, spanning slight to moderate ecological risk levels.

The spatial distribution of RI values in the study area was obtained through interpolation, as shown in Fig 4b. High ecological risk index values were mainly distributed in the bordering townships in the central part of the study area. Xiamaquan Township also exhibited a certain level of ecological risk, indicating some degree of soil pollution. In contrast, lower ecological risk levels were observed in the southern part of Hongtuliang Township bordering northern Xiaoshuanggou Township, as well as in the border areas between Jiashihe Township and Ximalin Township. Although Cd and Hg concentrations were lower than those of other elements, they contributed significantly to the ecological risk, likely due to their high toxicity coefficients. This suggests that ecological risk is influenced not only by element concentrations but also by their toxicity coefficients. Therefore, pollution control efforts should prioritize Cd and Hg in the study area.

#### 3.3.2. Concentration-orientated health risk assessment.

**Fig 5:**
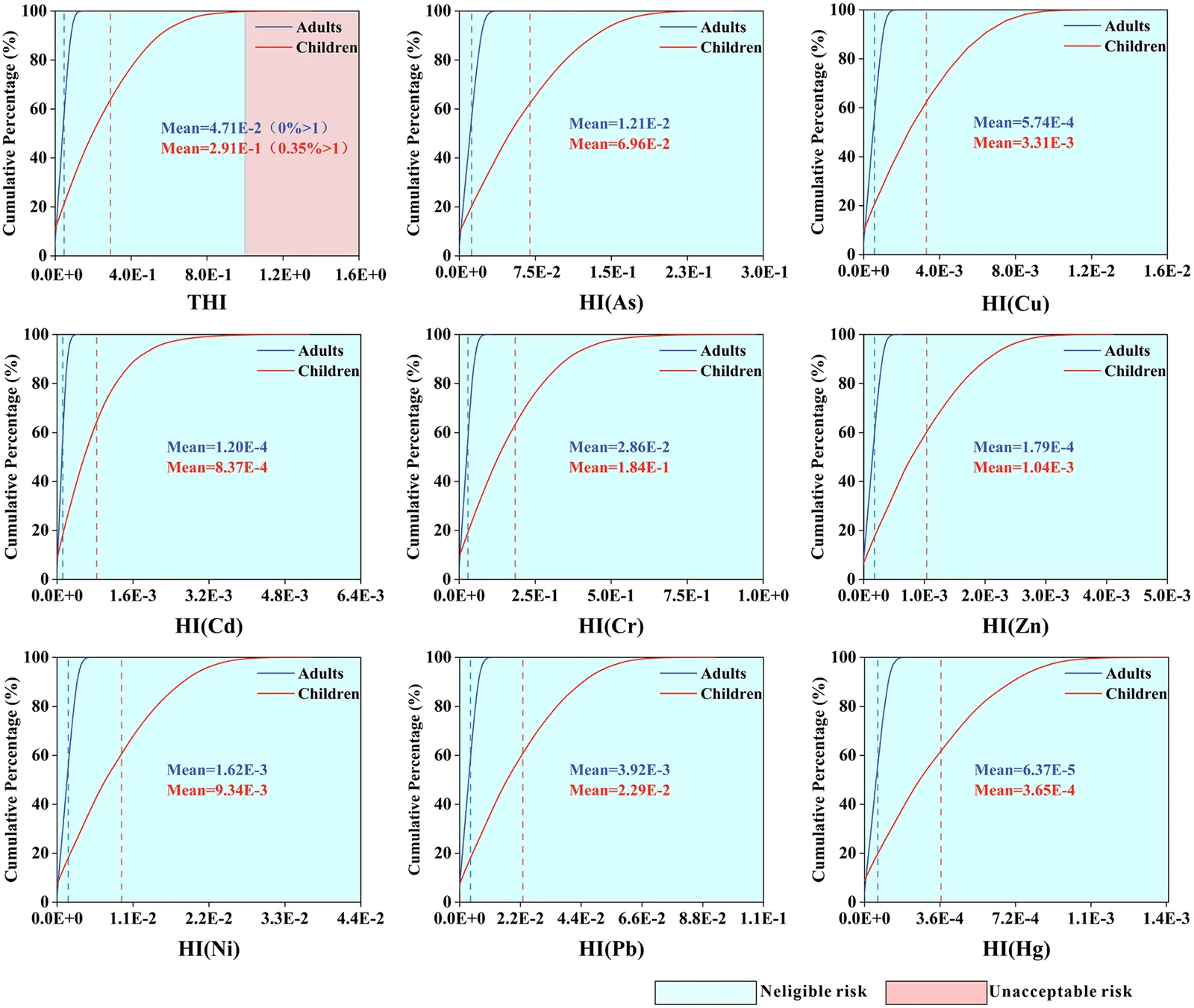
Health risk assessment of non-carcinogenic probability of PTEs.

In this study, a Monte Carlo simulation was employed to conduct a probabilistic health risk assessment of PTEs for both adult and pediatric populations. The probabilistic non-carcinogenic health risk results are presented in Fig 5. Children exhibited higher health risks than adults; however, no individual element posed a non-carcinogenic health risk to either population. The descending order of probabilistic non-carcinogenic health risk contribution for both adults and children was Cr> As> Pb > Ni > Cu > Zn > Cd > Hg. Based on the total hazard index (THI) values, the adult population remained within the safe range, indicating no non-carcinogenic risk. For children, 99.65% of THI values were below 1, also falling within the safe range, suggesting that the non-carcinogenic health risk is manageable.

**Fig 6:**
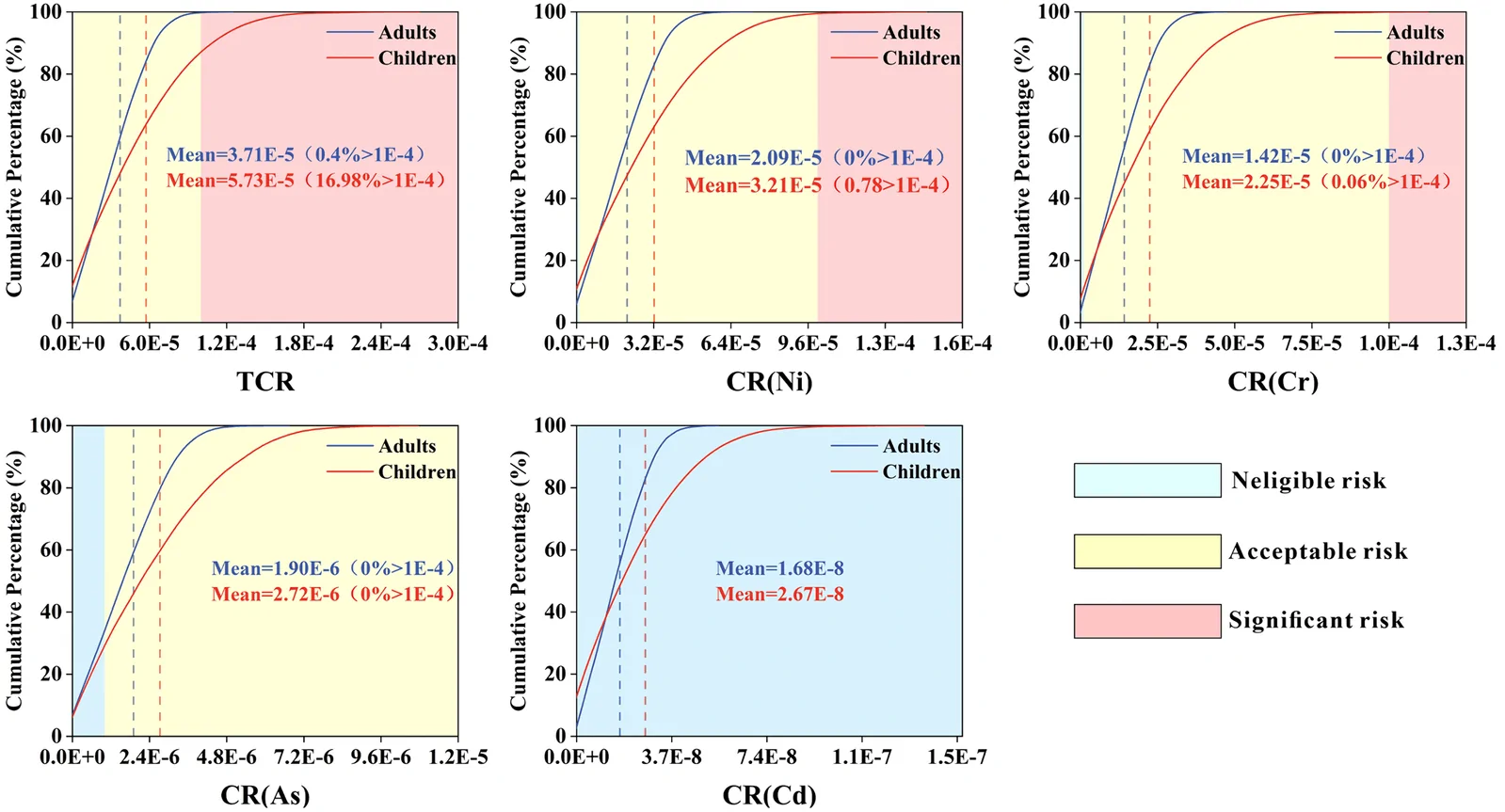
Health risk assessment of carcinogenic probability of PTEs.

Probabilistic carcinogenic health risks are shown in Fig 6. Only the element Cd is in the safe range of carcinogenic risk, and the average value of the carcinogenic risk of Cr, Ni, and As exceeds 10^−6^. As elements less than 10^−6^ accounted for 27.08% and 23.74% of the adult and child populations, respectively; The Ni element was greater than 10^−6^ but less than 10^−4^ in the adult population, while 0.78% of the children’s population had a cancer risk greater than 10^−4^, suggesting that the Ni element has a probabilistically significant cancer risk in the children’s population; The element Cr has a certain carcinogenic risk for adults and a significant carcinogenic risk of 0.06% for children, indicating that the carcinogenic risk of these three elements is not negligible. In total cancer risk (TCR) 0.4% of adults >10^−4^ had a significant cancer risk while 16.98% of children >10^−4^ had a significant cancer risk, indicating that children are more susceptible to cancer risk compared to adults.

#### 3.3.3. Source- orientated health risk assessment.

**Fig 7:**
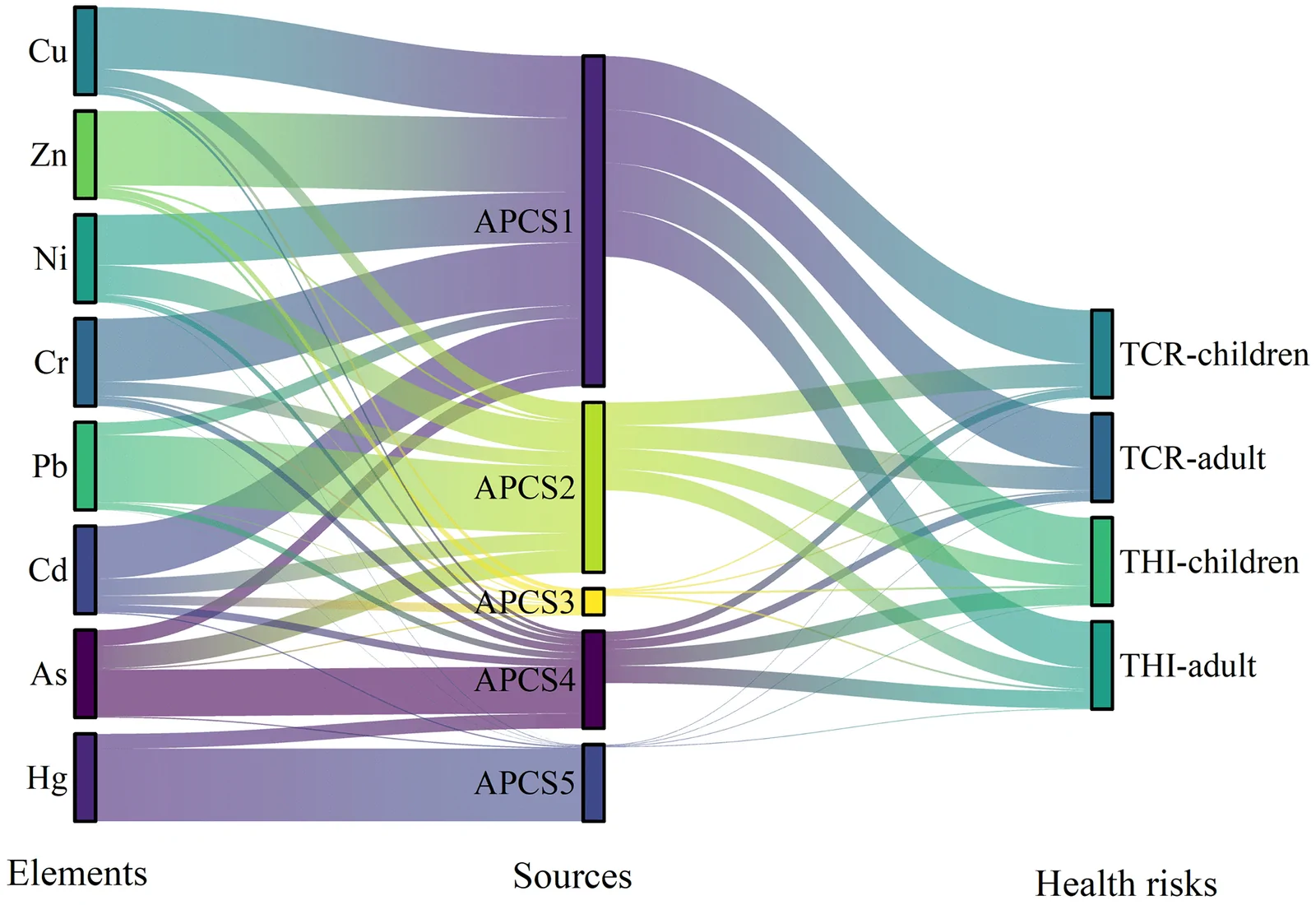
Sankey diagram of the relationship between PTEs, sources and health risks (width proportional to level of contribution).

In this study, a Sankey diagram was constructed to illustrate the relationships among soil potentially toxic element (PTE) concentrations, source contributions derived from the APCS-MLR model, and probabilistic health risks. As shown in Fig 7, the carcinogenic and non-carcinogenic risks from different sources exhibited similar trends for both adults and children, following the order: APCS1 > APCS2 > APCS4 > APCS3 > APCS5. Regarding non-carcinogenic risk, the contributions of different sources to health risks for adults (and children) were as follows: industrial sources accounted for 52.97% (54.08%), transportation sources for 24.16% (23.66%), natural sources for 19.78% (19.12%), agricultural sources for 2.29% (2.36%), and atmospheric sources for 0.80% (0.78%). For carcinogenic risk, the contributions were: industrial sources 60.95% (61.33%), transportation sources 26.49% (26.23%), natural sources 10.09% (9.96%), agricultural sources 1.81% (1.83%), and atmospheric sources 0.67% (0.65%).

Given that the overall non-carcinogenic health risk in the study area remains within a manageable range, the primary focus should be on carcinogenic health risks. Industrial and transportation sources were identified as the major contributors to carcinogenic risk, together accounting for the largest share of health risk. Therefore, risk control measures should be prioritized in areas with intensive industrial activities and transportation pollution, while minimizing the exposure frequency of the child population in these regions. Although natural sources ranked third in carcinogenic risk contribution, the dominant element from natural sources was As, which exhibits low enrichment in the study area. Consequently, the influence of natural sources on carcinogenic risk does not require significant attention.

## 4. Conclusion

This study quantified the risk status and sources of PTEs in regional surface soils using Monte Carlo health risk assessment and the APCS-MLR model. Key findings: average As, Pb, and Hg concentrations were below Hebei’s background values but showed notable accumulation. Five sources were identified: industrial (47.1%, dominant), transportation, agricultural, natural, and atmospheric deposition. Ecological risk assessment indicated low risks for As, Cr, Pb, Zn, Ni, and Cu, but high risks for Cd and Hg. Probabilistic health risks were greater for children than adults, with multi-element risks significantly exceeding single-element ones. Integrating probabilistic health risks with APCS-MLR enabled source-oriented risk assessment, identifying industrial and transportation sources as priority controls. This study establishes a priority control factor identification system for ecological and health risks, supporting regional environmental policymaking.Although this study has quantified the source contributions and risk levels of soil PTEs in the region, some uncertainties remain. Further research is required to investigate the impact of soil physical properties on soil toxicants (PTEs); a multi-index approach should be adopted to comprehensively assess regional pollution, whilst a long-term dynamic monitoring mechanism should be established to quantitatively evaluate the mitigating effect of policy implementation on the accumulation of soil toxicants, thereby providing evidence-based optimisation pathways for regional environmental management.

## Supporting information

S1 File S1 Table. Grading criteria for potential ecological risk factor and potential ecological risk index. S2 Table. Reference values of exposure parameters for human health risk assessment. S3 Table. Distributional characteristics of parameters in monte carlo simulations. S4 Table. Distribution settings for each parameter in the monte carlo simulation. S5 Table. Principal component analysis of soil trace elements in the study area. S6 Fig. The factor loading matrix after rotation.
(ZIP)
