# Supplementary material for: Source identification and risk assessment of potentially toxic elements in surface soil of townships in the northwest Hebei region
Source: PLoS One. 2026 Aug 28;21(8):e0353293. doi: 10.1371/journal.pone.0353293 (PMC13524235; doi:10.1371/journal.pone.0353293)
Supplement: Supplementary file 1 [file pone.0353293.s001.zip › S1 Table. Grading criteria for potential ecological risk factor and potential ecological risk index.docx]

### S1 Table . Grading criteria for potential ecological risk factor and potential ecological risk index

| Grade classification（E^i^_r_） | Grade classification（RI） | Pollution level |
| --- | --- | --- |
| $E_{r}^{i}$<40 | RI<150 | Low risk |
| 40≤$E_{r}^{i}$＜80 | 150≤RI<300 | Moderate risk |
| 80≤$E_{r}^{i}$＜160 | 300≤RI<600 | Considerable risk |
| 160≤$E_{r}^{i}$＜320 | RI≥600 | High risk |
| $E_{r}^{i}$≥320 | - | Very high risk |
