# Supplementary material for: Source identification and risk assessment of potentially toxic elements in surface soil of townships in the northwest Hebei region
Source: PLoS One. 2026 Aug 28;21(8):e0353293. doi: 10.1371/journal.pone.0353293 (PMC13524235; doi:10.1371/journal.pone.0353293)
Supplement: Supplementary file 1 [file pone.0353293.s001.zip › S2 Table. Reference values of exposure parameters for human health risk assessment..docx]

### S2 Table. Reference values of exposure parameters for human health risk assessment[1]

| Parameter | Parameter | Unit | adult | Children |
| --- | --- | --- | --- | --- |
| IngR | Ingestion rate of soil | mg/day | 100 | 200 |
| InhR | Inhalation rate of soil | m^3^/day | 14.5 | 7.5 |
| EF | Exposure frequency | day/year | 350 | 350 |
| ED | Exposure duration | year | 24 | 6 |
| BW | Body weight | kg | 56.8 | 15.9 |
| AT | Average exposure time (carcinogens) | day | 25550 | 25550 |
|  | Average exposure time ( non-carcinogens) | day | 8760 | 2190 |
| PEF | Particle emission factor | m^3^/kg | 1.36E+09 | 1.36E+09 |
| SA | Skin area available for soil contact | cm^2^ | 5075 | 2448 |
| AF | Soil-to-skin adherence factor | mg/cm^2^/day | 0.07 | 0.2 |
| ABS | Absorption factor | - | 0.001 | 0.001 |

# References

1.	Liu Z, Du Q, Guan Q, Luo H, Shan Y, Shao W. A Monte Carlo simulation-based health risk assessment of heavy metals in soils of an oasis agricultural region in northwest China. Sci Total Environ. 2023;857: 159543. doi:10.1016/j.scitotenv.2022.159543
