# Supplementary material for: Source identification and risk assessment of potentially toxic elements in surface soil of townships in the northwest Hebei region
Source: PLoS One. 2026 Aug 28;21(8):e0353293. doi: 10.1371/journal.pone.0353293 (PMC13524235; doi:10.1371/journal.pone.0353293)
Supplement: Supplementary file 1 [file pone.0353293.s001.zip › S3 Table. Distributional characteristics of parameters in monte carlo simulations.docx]

### S3 Table. Distributional characteristics of parameters in monte carlo simulations

| Heavy metals | *RfD*（mg$/(kg\cdot d)$） | | |  | *SF*（$\left( kg\cdot d \right)/mg$） | | |
| --- | --- | --- | --- | --- | --- | --- | --- |
|  | ingestion | inhalation | dermal  contact |  | ingestion | inhalation | dermal contact |
| Cr[1] | 3.00E-03 | 2.86E-05 | 6.00E-05 |  | 5.00E-01 | 4.20E+01 | 2.00E+01 |
| Ni[1] | 2.00E-02 | 2.06E-02 | 5.40E-03 |  | 1.70E+00 | 8.40E-01 | 4.25E+01 |
| As[2] | 3.00E-04 | 1.23E-04 | 3.00E-04 |  | 1.50E+00 | 1.51E+01 | 3.66E+00 |
| Hg[2] | 1.60E-04 | 8.60E-05 | 3.00E-04 |  | - | - | - |
| Cu[3] | 4.00E-02 | 4.02E-02 | 1.20E-02 |  | - | - | - |
| Zn[3] | 3.00E-01 | 3.00E-01 | 6.00E-02 |  | - | - | - |
| Cd[3] | 1.00E-03 | 1.00E-05 | 1.00E-05 |  | 5.10E-01 | 6.30E+00 | 2.00E+01 |
| Pb[3] | 3.50E-03 | 3.50E-03 | 5.25E-04 |  | 8.50E-03 | - | - |

# References

[1]	Z. Liu, Q. Du, Q. Guan, H. Luo, Y. Shan, W. Shao, A Monte Carlo simulation-based health risk assessment of heavy metals in soils of an oasis agricultural region in northwest China, Sci. Total Environ. 857 (2023) 159543. https://doi.org/10.1016/j.scitotenv.2022.159543.

[2]	C. Lin, W. Wang, G. Hu, R. Yu, H. Huang, D. Liao, Incorporating source apportionment and bioaccessibility into human health risk assessment of heavy metals in a soil-rice system in the Jiulong River basin, southeast China, Journal of Food Composition and Analysis 124 (2023) 105692. https://doi.org/10.1016/j.jfca.2023.105692.

[3]	L. Liu, Y. Li, X. Gu, R.X.S. Tulcan, L. Yan, C. Lin, J. Pan, Priority sources identification and risks assessment of heavy metal(loid)s in agricultural soils of a typical antimony mining watershed, Journal of Environmental Sciences 147 (2025) 153–164. https://doi.org/10.1016/j.jes.2023.11.007.
