# Supplementary material for: Source identification and risk assessment of potentially toxic elements in surface soil of townships in the northwest Hebei region
Source: PLoS One. 2026 Aug 28;21(8):e0353293. doi: 10.1371/journal.pone.0353293 (PMC13524235; doi:10.1371/journal.pone.0353293)
Supplement: Supplementary file 1 [file pone.0353293.s001.zip › S4 Table. Distribution settings for each parameter in the monte carlo simulation.docx]

### S4 Table. Distribution settings for each parameter in the monte carlo simulation[1]

| Parameter | Unit | Type |
| --- | --- | --- |
| IngR | mg/day | lognormal |
| InhR | m3/day | lognormal |
| EF | day/year | trigular |
| ED | year | uniform |
| BW | kg | normal |
| AT | day | point |
| PEF | m3/kg | point |
| SA | cm2 | lognormal |
| AF | mg/cm2/day | Beta |
| ABS | - | point |

# References

[1]	R. Ramesh, M. Subramanian, E. Lakshmanan, A. Subramaniyan, G. Ganesan, Human health risk assessment using Monte Carlo simulations for groundwater with uranium in southern India, Ecotoxicology and Environmental Safety 226 (2021) 112781. https://doi.org/10.1016/j.ecoenv.2021.112781.
