# Supplementary material for: Source identification and risk assessment of potentially toxic elements in surface soil of townships in the northwest Hebei region
Source: PLoS One. 2026 Aug 28;21(8):e0353293. doi: 10.1371/journal.pone.0353293 (PMC13524235; doi:10.1371/journal.pone.0353293)
Supplement: Supplementary file 1 [file pone.0353293.s001.zip › S5 Table. Principal component analysis of soil trace elements in the study area.docx]

### S5 Table. Principal component analysis of soil trace elements in the study area

| PCA | Initial eigenvalue | | |  | Extract the sum of the squares of the loads | | |  | Rotational load square sum | | |
| --- | --- | --- | --- | --- | --- | --- | --- | --- | --- | --- | --- |
|  |  |  |  |  |  |  |  |  |  |  |  |
|  | Total | Var(%) | Acc(%) |  | Total | Var(%) | Acc(%) |  | Total | Var(%) | Acc(%) |
| 1 | 3.870 | 48.38 | 48.38 |  | 3.870 | 48.38 | 48.38 |  | 3.294 | 41.17 | 41.17 |
| 2 | 1.398 | 17.48 | 65.86 |  | 1.398 | 17.48 | 65.86 |  | 1.080 | 13.50 | 54.67 |
| 3 | 0.952 | 11.91 | 77.76 |  | 0.952 | 11.91 | 77.76 |  | 1.040 | 13.00 | 67.67 |
| 4 | 0.659 | 8.24 | 86.00 |  | 0.659 | 8.24 | 86.00 |  | 1.019 | 12.73 | 80.41 |
| 5 | 0.556 | 6.95 | 92.95 |  | 0.556 | 6.95 | 92.95 |  | 1.004 | 12.55 | 92.95 |
| 6 | 0.252 | 3.15 | 96.11 |  |  |  |  |  |  |  |  |
| 7 | 0.195 | 2.43 | 98.54 |  |  |  |  |  |  |  |  |
| 8 | 0.117 | 1.46 | 100.00 |  |  |  |  |  |  |  |  |
