# Supplementary material for: Source identification and risk assessment of potentially toxic elements in surface soil of townships in the northwest Hebei region
Source: PLoS One. 2026 Aug 28;21(8):e0353293. doi: 10.1371/journal.pone.0353293 (PMC13524235; doi:10.1371/journal.pone.0353293)
Supplement: Supplementary file 1 [file pone.0353293.s001.zip › S6 Fig. The factor loading matrix after rotation.docx]

### S6 Fig. The factor loading matrix after rotation
